# Optimization of Microwave-Assisted Water Extraction to Obtain High Value-Added Compounds from Exhausted Olive Pomace in a Biorefinery Context

**DOI:** 10.3390/foods11142002

**Published:** 2022-07-06

**Authors:** Irene Gómez-Cruz, María del Mar Contreras, Inmaculada Romero, Eulogio Castro

**Affiliations:** 1 Department of Chemical, Environmental and Materials Engineering, Universidad de Jaén, Campus Las Lagunillas, 23071 Jaén, Spain; igcruz@ujaen.es (I.G.-C.); iromero@ujaen.es (I.R.); ecastro@ujaen.es (E.C.); 2 Centre for Advanced Studies in Earth Sciences, Energy and Environment (CEACTEMA), Universidad de Jaén, Campus Las Lagunillas, 23071 Jaén, Spain

**Keywords:** exhausted olive pomace, green extraction, microwave-assisted water extraction, hydroxytyrosol, triterpenic acids

## Abstract

Microwave-assisted water extraction (MAWE) was evaluated to obtain the valuable bioactive compounds hydroxytyrosol and mannitol from exhausted olive pomace (EOP). The influence of the operational parameters solid loading (3–15%, *w*/*v*), temperature (40–100 °C), and extraction time (4–40 min) was studied using an experimental design. The optimized conditions maximizing their joint extraction were 12% *w*/*v* solid loading, 100 °C temperature, and 16 min. It was possible to solubilize 5.87 mg of hydroxytyrosol/g EOP and 46.70 mg mannitol/g EOP. The extracts were also further characterized by liquid chromatography–mass spectrometry, which detected other hydroxytyrosol derivatives such as oleacein, verbascoside, and oleuropein. Moreover, the applied MAWE conditions promoted the co-extraction of proteinaceus material, which was also evaluated. In order to carry out an integral valorization of this waste, the extracted EOP solid was further evaluated chemically and microscopically before recovering the bioactive triterpenes. In particular, maslinic acid and oleanolic acid were obtained, 9.54 mg/g extracted solid and 3.60 mg/g extracted solid, respectively. Overall, MAWE can be applied as a first stage in the fractionation of EOP to support its valorization in a biorefinery framework.

## 1. Introduction

Hydroxytyrosol is considered a valuable bioactive compound because of its antioxidant activity and potential health benefits. Hydroxytyrosol and hydroxytyrosol-rich extracts can be applied in foods to promote their preservation [1] and as active ingredients in films for food packaging applications by adding antioxidant properties [2]. Its safety has been reported EFSA et al. [3]. Moreover, as a functional ingredient, it could prevent and treat different diseases [4]. Hydroxytyrosol can be produced by chemical and biological synthesis [5], but its recovery from natural sources (e.g., olive fruit and derived biomasses) is in line with the increase in demand for natural food additives as consumers seek products with ingredients of natural origin [6]. Particularly, the exhausted olive pomace (EOP) is the final residual solid generated after subjecting olive pomace to a drying process and a solid-liquid extraction with hexane to extract the residual olive oil. It is produced in the olive oil production chain in some Mediterranean countries and contains hydroxytyrosol [7]. Thereby, it is an interesting bioresource to be exploited.

Furthermore, recent studies suggest that EOP is not only a cheap source of hydroxytyrosol but also presents mannitol and triterpenic acids [8,9,10]. Particularly, mannitol is an authorized sweetener (E421) widely used because of its low calorie content and it is the active ingredient of some drugs and cosmetics [11]. Besides being used as an osmotic diuretic, mannitol also has the clinical potential for treating diseases such as Parkinson’s disease, cystic fibrosis, migraines, etc. [12]. Similar to hydroxytyrosol, mannitol can also be obtained by chemical and biotechnological approaches [12]. Nevertheless, its extraction from natural sources is desirable and olive-derived biomasses can be an interesting option. Therefore, the green extraction of these biocompounds is attractive for industrial applications of these underused bioresources, whose valorization would contribute to economic and environmental sustainability.

Microwave-assisted extraction (MAE) can be applied as an energy efficient technique for extracting bioactive compounds from biomass. It is explained by its capacity for heating a matrix both internally and externally with no thermal gradient. Microwaves promote cell alteration and improve the recovery of the compounds of interest [13]. This allows for reducing extraction times, energy consumption, and solvent requirement [14]. This fact makes MAE an environmentally friendly technology, especially if green solvents are applied.

Although this technology is not well developed at a large scale, microwaves at industrial or pilot scale can process around 100 kg of biomass [15]. In the case of future deployment, studies at the laboratory scale can help to understand the effect of the operational parameters, which depend on the biomass and the biocompound [15,16]. They can also provide information about the bioactive content and profile that can be obtained using this technology and the chemical composition changes of the biomass to plan its complete exploitation in a circular scenario.

For the first time, the co-extraction of hydroxytyrosol and mannitol from the untapped bioresource EOP by microwave-assisted water extraction (MAWE) in a biorefinery context was studied and optimized. The effect of MAWE on the extraction of proteinaceous material was also evaluated, and as well on the chemical and microscopical characteristics of the EOP. Moreover, a second extraction step allowed for the recovery of triterpenic acids, which also have multiple beneficial health and pharmacological properties. Overall, this study adds value to the pomace olive oil production process to move it towards the goal of zero waste based on a cascading extraction of biocompounds.

## 2. Materials and Methods

### 2.1. Biomass

The pelletized EOP (~6.5% moisture content) was supplied by an olive pomace oil extraction factory (Espuny SA, Jaén, Spain) and then it was milled in the laboratory (~1 mm). The raw EOP was analyzed following the analytical methods of the National Renewable Energy Laboratory (NREL, Golden, CO, USA) [17] and its chemical composition (dry weight) was 9.7% cellulose, 10.9% hemicellulose, 21.8% lignin, 41.8% extractives, and 6.4% ash [18].

### 2.2. Methodology

**Figure 1:**
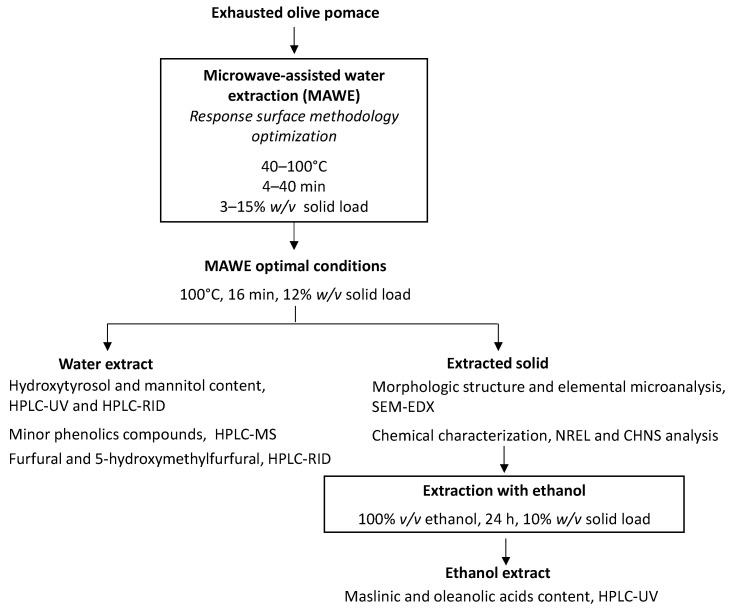
Experimental methodology followed in the present study.

MAWE of bioactive compounds from EOP was studied. The effect of the most important variables (solid loading, temperature, and time extraction) was analyzed using the response surface methodology (RSM). Once these parameters were optimized, the aqueous extract obtained at optimized conditions was further characterized to profile its phenolic compounds and to measure its protein content. The presence of undesirable compounds furfural and hydroxymethylfurfural was also determined. Moreover, the effect of the MAWE conditions on the biomass was evaluated chemically and microscopically for future biorefinery applications. Finally, in order to recover maslinic and oleanolic acids from the extracted EOP solid, a second extraction step was performed using ethanol as the solvent (Figure 1).

### 2.3. Experimental Design and Optimization of MAWE

MAWE was carried out with an Anton Paar Monowave 400 microwave system (Graz, Austria) using a high-pressure 30 mL reactor that can reach up to 300 °C, 850 W, and 30 bars. The biomass was suspended in distilled water, and the suspension was continuously stirred with a magnetic bar. The slurry obtained after irradiation was filtered under vacuum to separate the aqueous extract from the solid fraction (extracted EOP solid). The extracts were filtered (0.45 μm syringe filters) (SinerLab Group, Madrid, Spain) for further analysis. Extraction yields were determined gravimetrically after the extracts were dried in an oven (Memmert, Schwabach, Germany) at 105 °C until constant weight. Extraction yields were referred to initial dry biomass and were expressed as g extract/100 g EOP. In addition, the extracted EOP solid was oven-dried (40 °C) and stored in hermetically sealed bags.

The extraction tests were conducted following a Box–Behnken experimental design to analyze the impact of the factors, temperature (40–100 °C), extraction time (4–40 min), and solid loading (3–15%, *w*/*v*), on the content of phenols, hydroxytyrosol and mannitol, and antioxidant activity (Table 1). Thus, 17 experiments were performed in a random order. The central point was performed five times to evaluate the precision of the model. The response surface methodology (RSM) was applied using the software Design-Expert^®^ v8.0.7.1 (Stat-Ease, Inc., Minneapolis, MN, USA), and second-degree polynomial equations for each response variable were obtained according to the following equation:

(1) $$y_{j}=\beta_{0}+\overset{3}{\underset{i=1}{\sum }}\beta_{i}x_{i\;}+\sum \overset{3}{\underset{i<j=1}{\sum }}\beta_{ij}x_{i}x_{j}+\overset{3}{\underset{i=1}{\sum }}\beta_{ii}x_{i}^{2}$$

where *y* is the response variable, *β*_0_, *β_i_*, *β_ij_*, and *β_ii_* are the regression coefficients calculated by the least-squares method, and *x_i_* and *x_j_* are the factors (coded from −1 to 1). Using this software, the optimal conditions were obtained after analyzing the response surfaces of each variable studied. These conditions were then tested experimentally (n = 5) to check the validity of the optimization. The five extracts and the resulting extracted EOP solids were characterized as in the following sections. These solids were also subjected to ethanolic extraction to recover triterpenic acids (Section 2.6).

### 2.4. Characterisation of the MAWE Extracts

#### 2.4.1. Total Phenolic Content (TPC) and Antioxidant Properties of the Extracts

The Folin–Ciocalteu colorimetric test was applied to determine the TPC according to Gómez-Cruz et al. [7]. In a test tube, 0.3 mL of diluted extract (or solvent for blanks), 3 mL of Folin–Ciocalteu reagent 2 M (Sigma-Aldrich, St. Louis, MO, USA), and 2 mL of Na_2_CO_3_ (Sigma-Aldrich) solution (10% *w*/*v*) were added. Then, the mixture was agitated in vortex and 300 μL of the sample was added to a microwell plate. After 1 h in the dark at room temperature, the absorbance was measured at 760 nm in a Bio-Rad iMark reader (Hercules, CA, USA). Gallic acid was used to build a reference standard curve, and the results were expressed as concentration (g of gallic acid equivalents (GAE)/L) and referred to the raw material (mg GAE/g EOP).

The antioxidant capacity of the EOP extracts was carried out using the ferric reducing power assay (FRAP) and the ABTS™ radical scavenging assays according to Gómez-Cruz et al. [7]. In the FRAP assay, FRAP reagent was first prepared by mixing 300 mM acetate buffer (pH = 3.6), a solution of 10 mM TPTZ in 40 mM HCl, and 20 mM FeCl_3_∙6H_2_O in distilled water in the ratio 10:1:1. Then, 100 µL of the diluted extract (or solvent for blanks) was added to 3 mL of FRAP reagent. Finally, after 6 min in the dark and at room temperature, the absorbance was measured at 593 nm. In the ABTS assay, a stock solution of ABTS (7 mM) was diluted with 2.45 mM potassium persulfate with phosphate buffer (PBS) (pH = 7.4) to an absorbance of 0.7 at 734 nm. Then, 3 mL of TEAC reagent was added to 30 µL of the diluted extract and, after 6 min in the dark, the absorbance at 734 nm was measured. All the measurements were performed in triplicate in the aforementioned device and the results were obtained as mg Trolox Equivalents (TE)/g EOP. All reagents were procured from Sigma-Aldrich.

#### 2.4.2. Liquid Chromatography of Phenolic Compounds and Mannitol

The phenolic compounds were profiled in the aqueous extracts by HPLC with a diode array detector (Shimadzu, Kyoto, Japan) according to Lama-Muñoz et al. [19]. A reversed-phase C18 column (250 mm × 4.6 mm), type BDS HYPERSIL 5 µm (Thermo Fisher Scientific Inc., Waltham, MA, USA), and a ternary solvent gradient consisting of 0.2% orthophosphoric acid-water (A), methanol (B) (PanReac AppliChem, Barcelona, Spain) and acetonitrile (C) (PanReac AppliChem) were used. The initial composition was 96/2/2 (*v*/*v*/*v*), and then the elution gradient was as follows: B and C changed from 2% to 25% in 40 min, 25% to 30% in 5 min, 30% to 50% in 15 min, isocratic to 50% for 8 min, and then 50% to 2% in 4 min. The elution flow rate was 1 mL/min, the oven temperature was set at 30 °C, and the volume of sample injected was 20 μL. The hydroxytyrosol content was determined by comparison with its commercial standard (Extrasynthese, Genay, France) at 280 nm (*y* = 19,113*x* − 15,977; R^2^ = 0.999). The data were expressed as g hydroxytyrosol/L and mg hydroxytyrosol/g EOP.

In addition, minor phenolic compounds were characterized following the methodology previously described by Contreras et al. [10] using HPLC (1100 series, Agilent Technologies, St. Clara, CA, USA) coupled to an Esquire 6000 ion trap (Bruker, Bremen, Germany) via an electrospray ionization source. Phenolic compounds were eluted at 0.35 mL/min using Milli-Q^®^ water and formic acid (0.1%, *v*/*v*) as solvent A and acetonitrile and formic acid (0.1%, *v*/*v*) as solvent B. A Kinetex core-shell C18 column (2.1 × 50 mm, 2.7 μm) (Phenomenex, Barcelona, Spain) was used and a linear gradient of solvent B in A [10]. MS spectra were recorded in the mass-to-charge (*m*/*z*) range from 100 to 1200 in the negative ionization mode. Auto MS^2^ analyses were carried out at 0.6 V and acquired in the latter range, averaging at least two spectra. The data were processed using Bruker DataAnalysis (version 4.0). According to a previous study [10], HPLC (1200 series, Agilent Technologies) coupled to quadrupole-time-of-flight (QTOF) (Agilent 6530B) mass spectrometry (MS) was also used to obtain mass accurate data and generate the molecular formula of the compounds. The interface was an electrospray ionization source and the analyses were performed in the negative ionization mode in the *m*/*z* range 60–1200 Da. A continuous infusion of trifluoroacetic acid ammonium salt (*m*/*z* 112.9856) and hexakis 1H,1H,3H–tetrafluoropropoxy) phosphazine (*m*/*z* 1033.9881) (Agilent Technologies) was carried out for mass correction. The column and gradient employed was as described above. MassHunter Qualitative Analysis B.06.00 (Agilent Technologies) was used for data analysis.

Mannitol (Sigma-Aldrich) was quantified by HPLC (2695 model; Waters corporation, Milford, MA, USA) equipped with refractive index detection (RID) from Agilent Technologies. Samples were previously conditioned with ion exchange resins (Microionex MB200, Rohm Haas, Denmark) to remove impurities and then filtered through 0.45 µm nylon membranes. A carbohydrate column (CARBOSep CHO-782 Pb; Transgenomic, Inc., Omaha, NE, USA) operating at 70 °C and Milli-Q^®^ water as mobile phase was used. The flow rate and injection volume were 0.6 mL/min and 20 μL, respectively. The data were given as concentration (g mannitol/L) and referred to the raw material (mg mannitol/g EOP).

#### 2.4.3. Analysis of the Protein

The solubilized protein was determined by the Bradford kit assay from Bio-Rad. The protein content in the EOP extracts was measured by spectrophotometry at 595 nm with the cited microplate absorbance reader. Bovine serum albumin (BSA) was employed as a reference for comparison. All analyses were conducted in triplicate, and the results referred to the raw material (mg BSA equivalents/g EOP).

#### 2.4.4. Analysis of Furfural and Hydroxymethylfurfural

A 1260 series HPLC (Agilent Technologies) device connected to a RID detector was used to measure furfural and hydroxymethylfurfural content in the extracts. An ICSep ICE-COREGEL 87 H3 ICSep column (Transgenomic, Inc.) was used, the temperature was set at 65 °C, and the flow rate of the mobile phase (5 mM sulfuric acid) was 0.6 mL/min.

Using the external calibration method, the curves obtained were furfural (*y* = 4.28 ×∙10^5^*x* − 8.95 ×∙10^4^; R^2^ = 0.9998) and hydroxymethylfurfural (*y* = 3.64 ×∙10^5^*x* − 2.45 ×∙10^4^; R^2^ = 0.9996). Both compounds were procured from Sigma-Aldrich. The data were expressed as g/L and mg/g EOP.

### 2.5. Characterization of the Extracted EOP Solid after MAWE

The extracted EOP solid obtained after the application of MAWE under optimized conditions was chemically characterized according to the standard NREL [17]. In this methodology, the first step consists of determining the moisture and ash content of the biomass. In the next step, the non-structural components of biomass (extractives) was determined by means of a two-step Soxhlet extraction: a first step with water (aqueous extractives), and, in a second step, ethanolic extraction (ethanolic extractives) was performed. This extraction is prior to the analysis of the rest of the components of the raw material, in order to avoid interferences in the case of materials with a high content of extractives, which is the case of EOP. The next step consists of a two-step acid hydrolysis, first with concentrated H_2_SO_4_ (72% *v*/*v*) and then with diluted H_2_SO_4_ (3–4% *v*/*v*), in order to solubilize all the structural carbohydrates for their determination. The hydrolysates obtained were analyzed by HPLC-RID (as for mannitol analysis) to determine the concentration of glucose, xylose, galactose, arabinose, and mannose, from which the polysaccharide content (cellulose and hemicellulose) was calculated. In addition, the acetyl group content of the hydrolysate was quantified by HPLC-RID and the soluble acid lignin content by spectrophotometry at 205 nm. Finally, the acid-insoluble lignin and acid-insoluble ash contents (575 °C, 4 h) were determined by weighing the solid resulting from the acid hydrolysis.

The elemental analysis of the extracted EOP solid was also determined using a TruSpec Micro device (Leco, St. Joseph, MI, USA). For this purpose, 2 mg of sample was subjected to total combustion in a furnace. Then, using different detectors, the amount of carbon, hydrogen, nitrogen, and sulfur was quantified.

In addition, the raw milled EOP and the extracted EOP solid obtained under optimal MAWE conditions were analyzed by field emission scanning electron microscopy (SEM) (Merlin Carl Zeiss equipment) (Carl Zeiss, Oberkochen, Germany) coupled to an energy dispersive X-ray spectrometry (EDX) detector (Oxford Inca Energy 350X-MAX 50) (Oxford Instruments, Abingdon, UK). In the case of the elemental microanalysis, the samples were metalized with carbon at 20 kV voltage. All samples were examined under high vacuum conditions.

### 2.6. Extraction and Analysis of Triterpenic Acids

The initial milled EOP (in triplicate) and the five EOP solids resulting after MAWE under optimized conditions were extracted with absolute ethanol (VWR Chemicals; Radnor, PA, USA) at room temperature for recovering triterpenic acids [8]. Extraction was performed on a rotary shaker (INFORS HT Ecotron, Surrey, UK) at room temperature (24 h, 150 rpm) and 10% solid loading (*w*/*w*). Each sample was centrifuged (MicroCen 16, Herolab, Wiesloch, Germany) and filtered with a syringe filter (nylon, pore size 0.45 μm) (SinerLab Group). The extraction yield of all the ethanolic extracts was determined as described in Section 2.3. The content of triterpenic acids in the extracts was determined by HPLC (as in Section 2.4.2) at 210 nm through curves for maslinic acid (Extrasynthese) (*y* = 8118*x* + 123,127; R^2^ = 0.997) and oleanolic acid (Extrasynthese) (*y* = 10,717*x* + 48,413; R^2^ = 0.999). The results were expressed as concentration (g/L) and referred to the raw material (mg/g dry weight).

### 2.7. Statistical Analysis

F-test and *t*-test were performed for two means comparison using Excel Office 2019 (Microsoft, Albuquerque, NM, USA). The response surface methodology analysis was carried out with Design-Expert^®^ v8.0.7.1 software, as commented before.

## 3. Results

### 3.1. MAWE Extraction of EOP and Model Equations

Water was the solvent selected because it has been previously tested and shown to be a green solvent capable of extracting phenolic compounds from EOP [7]. In addition, water presents a high dielectric constant and an intermediate dissipation factor (or dielectric loss tangent) that allow heat to be distributed throughout the matrix when using microwave radiation [16].

The experimental results for each response variable (extraction yield, content, and concentration of total phenolics, hydroxytyrosol and mannitol, and antioxidant activity) obtained in the BBD are shown in Table 1. The variability found in these results highlights the fact that the values of the response variables highly depend on the MAWE conditions applied and RSM can be of help in looking for their optimization. In fact, temperature, time, and solid loading are crucial parameters that need to be optimized for successful solubilization of bioactive compounds and carbohydrates by MAWE [16].

In this line, Table 2 shows the parameters of the second-degree polynomial equations, which describe the relationship between the operational parameters (temperature, time, and solid loading) and the response variables, as well as the statistical results for these models. The ANOVA (*p*-values < 0.05), the R^2^ values higher than 0.9595, and the CV values lower than 5% indicate that the models were adequate in adjusting the results and explaining the influence of the factors on the selected responses. Furthermore, the models showed no lack of fit (*p*-value > 0.09, Table 2), indicating that the dispersion of the experimental results was negligible.

### 3.2. Effect of the MAWE Parameters on the Yield and Biocompounds

**Figure 2:**
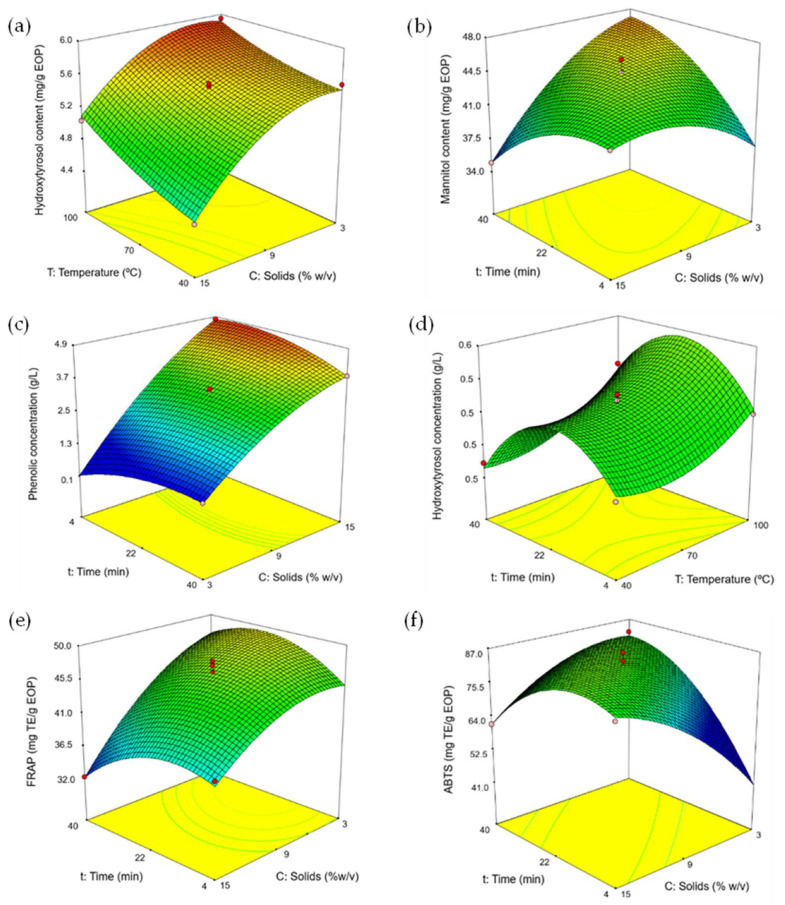
Response surfaces for the Box–Behnken design of: (**a**) hydroxytyrosol content, (**b**) mannitol content, (**c**) phenolic concentration, (**d**) hydroxytyrosol concentration, (**e**) FRAP assay, and (**f**) ABTS assay. In each figure, the third factor was fixed at the intermediate level: 9% solid loading (**d**), 22 min (**a**), or 70 °C (**b**,**c**,**e**,**f**).

According to the mathematical models, all the factors, solid loading, extraction time, and temperature showed an influence on the response variables but with a different impact. As shown in Table 2 (Equation (2)), the extraction yield depends mainly on the temperature and solid loading, with the linear terms of both parameters having a similar but opposite influence. A similar behavior was observed in the models obtained for the TPC and the hydroxytyrosol and mannitol content (Table 2, Equations (4), (6), and (8)). In general, a positive effect of the temperature was observed on the three responses. Moreover, the quadratic terms of the time and the solid loading also had a high relevance and indicate that a maximum was reached. As an example, Figure 2a represents the response surface for the hydroxytyrosol content as a function of temperature and solid loading at 22 min. There was a negative interaction between the time and the solid loading, indicating that as time and solid loading simultaneously increase, a lower extraction of the bioactive compounds occurs. As another example, Figure 2b shows the influence of the extraction time and solid loading on the mannitol content for a temperature of 70 °C.

Concerning the responses obtained by the model for the concentration of phenolic compounds, hydroxytyrosol, and mannitol in the liquids, they presented similar trends and showed that all the factors have an influence on them (Table 2, Equations (3), (5), and (7)). Nonetheless, the linear term of the solid loading was the most significant and had a positive effect on all three responses. As an example, Figure 2c shows the response for the concentration of phenolic compounds as a function of the solid loading and the time with the temperature set at 70 °C. Alternatively, Figure 2d shows the influence of the temperature and extraction time on the concentration of hydroxytyrosol at 9% *w*/*v* solid loading, showing that a maximum is reached for the time at intermediate values.

Previous studies suggest that in MAWE, the extraction performance is enhanced due to the effective heating effect generated as a result of the dipole rotation of water molecules and the ionic conductions of dissolved ions. At high temperatures, the viscosity and interfacial surface tension of water are reduced, which favor its diffusion through the biomass matrix and the solubility of the biocompounds [16]. Time also positively influences the extraction efficiency, but long times, especially at high temperatures, can cause degradation and oxidation of the compounds of interest [13]. This would explain the need to generally apply intermediate values of extraction time, as observed in previous figures, and the negative interaction between extraction time and temperature. Therefore, short extraction times should be applied in MAE, which is one of the advantages attributed to this technique [20]. It can be noted that the studied bioactive compounds are quite resistant to the temperature, at least in the domain used in this research, as shown by their ability to resist 100 °C for a certain time. It has been suggested that many phenols are thermal resistant, even up to temperatures of 100–125 °C, with those that have fewer hydroxyl-type substituents being more resistant [21]. In the case of mannitol, under subcritical conditions, it was stable up to around 100 °C [22].

It is crucial to optimize the solid loading to reduce the solvent volume requirement, but favoring adequate microwave irradiation into the suspension [16]. It was observed that the use of high solid loadings specially decreased the efficiency of extraction (per g of EOP) of phenolic compounds, including hydroxytyrosol, (e.g., Figure 2a). Previous studies have shown that, besides the effect of the irradiation, lower solid-to-liquid ratios can increase the contact surface between the biomass matrix and the solvent favoring the mass transfer, while adsorption phenomena at higher ratios can contribute reducing the efficiency of extraction [21].

The three studied parameters also had an influence on the antioxidant capacity of the extracts as determined by the FRAP and ABTS methods (Table 2, Figure 2e,f). FRAP followed a similar trend for the extraction yield, TPC, and the content of hydroxytyrosol and mannitol. In addition, negative interactions between the factors were observed (Table 2), indicating that when these parameters increase simultaneously, a lower antioxidant capacity is achieved. In this sense, the extraction is more effective when higher temperatures and shorter extraction times are used. Moreover, this avoids thermal degradation in MAE, which correlates with the previous results.

### 3.3. Optimization of the Extraction Parameters

The optimization of MAWE is crucial since the optimal operational conditions depend on the biocompound object of extraction. These conditions are also influenced by the biomass properties (e.g., moisture, particle size, etc.) and the extractive agent [15,16]. In the present study, MAWE of EOP was optimized by maximizing simultaneously the nine responses. According to the BBD, the optimal conditions were found at 100 °C, 16 min extraction time, and 12% *w*/*v* solids. Then, the optimal conditions were reproduced at the laboratory, and the results were similar to the theoretical data predicted by the model (error < 5% in all cases) (Table 3).

MAWE was efficient for recovering phenolic compounds from EOP. Compared to the conventional hydrothermal extraction of the same raw material at 85 °C using a water bath for heating, MAWE yielded the same concentration but required a short time, 16 min vs. 90 min [7]. In TPC in the EOP extracts compares favorably with the results reported by Tapia-Quirós et al. [23] about the microwave assisted extraction of olive pomace. It required the use of 50% ethanol as solvent at 90 °C at 5 min. Differences in the biomass composition, solvent, and device may explain the differences in the results and conditions applied. Singh et al. [24] observed that the dielectric properties of alcoholic aqueous solutions depend on the temperature, methanol percentage, and presence of biomass matrix at 2.45 GHz and 0.915 GHz microwave frequency, and it has an impact on the extraction.

Regarding mannitol, the amount extracted from EOP by MAWE under optimized conditions, 46.7 mg/g EOP (Table 3), was higher than that obtained with water as a solvent using a Soxhlet extractor overnight (40 mg/g EOP) [11] and in a bath at 85 °C for 90 min (45 mg/g EOP) [7] or at 100 °C for 30 min (39 mg/g EOP) [25]. In addition, it is worth noting that these amounts of mannitol extracted from EOP are in the range of the values obtained from olive leaves, which are considered a rich source of mannitol [19]. Microwave radiation penetrates into the biomass at the speed of light, producing rapid and uniform heating due to ionic conduction and dipole rotation at molecule level. It shortens the extraction time compared with conventional heating methods, where the transference of heat is produced by convection and conduction [16]. Although the content of bioactive compound on olive fruit derived byproducts depends on a number of factors (olive cultivar, olive ripening degree, primary and time of olive oil processing within the campaign, type of byproduct, etc.) [5,9,10,25], the latter comparison highlights that EOP can be applied to recover hydroxytyrosol and mannitol using MAWE.

### 3.4. Profiles and Standardization

The HPLC analysis at 280 nm of the EOP extracts obtained by MAWE under different conditions showed that hydroxytyrosol was the main phenolic compound identified (as an example, Figure S1). This is in agreement with previous works with the same feedstock extracted by hydrothermal treatment at 85 °C [7] and ultrasound-assisted water extraction [8].

The hydroxytyrosol content ranging from 3.78 to 6.05 mg/g EOP was determined in the extracts (Table 1). In addition, the minor phenolic compounds were characterized according to the methodology of previous studies based on HPLC-MS through electrospray ionization (negative mode) [8,10]. Basically, the compounds were characterized on the basis of their retention time, *m*/*z*, and fragmentation pattern, which were provided by using an ion trap (Table S1). These data were compared to those of the compounds previously characterized in the latter works, using the same methodology, and literature to provide the compound assignment. Additionally, QTOF analysis was used to obtain the molecular formula and confirm the characterization. It is worth noting that the aqueous EOP extract presents several hydroxytyrosol derivatives, including other interesting bioactive compounds such as oleacein, verbascoside, and oleuropein. Phenolic acids, flavonoids, and elenolic acid derivatives were also detected (Table S1). A similar qualitative profile was obtained by ultrasound-assisted extraction [8], suggesting that MAWE can be applied as an alternative technology to recover these biomolecules from EOP.

### 3.5. Protein Solubilized in the EOP Extracts

Efficient extraction of proteins from biomass increases the value of production chains. Due to their composition, proteins are of interest in the chemical and food industries [26]. The protein solubilized in the aqueous extracts was determined, and the effect of the MAWE experimental parameters on the protein solubilization was evaluated. In the BBD experiments, the protein concentration of the extracts ranged from 0.19 g/L (run 2) to 2.56 g/L (run 16), corresponding to 5.76 mg BSA/g EOP and 17.59 mg BSA/g EOP, respectively (Table S2). A significant influence of the solid loading affecting the protein concentration in the extracts was observed, while a certain influence of the temperature was observed when the data were expressed as protein content (referring to the raw material) (Table S3). This means that, in general, a higher protein content is obtained when higher temperatures are used. Moreover, the protein concentration in the aqueous extract obtained under optimized conditions was 1.98 ± 0.14 g/L, corresponding to 15.88 ± 0.84 mg/g EOP. This protein concentration makes sense compared to the BBD results because it was obtained at an intermediate solid loading but at the highest temperature (12% *w*/*v*, 100 °C, 16 min).

Previous studies have shown that it is easier to extract the proteinaceous material from EOP than from olive leaves or olive tree pruning using hot water [11]. This can explain the partial solubilization of the EOP protein observed in this work. Moreover, other studies have proposed novel extraction methods, such as pulsed electric field and high pressure, to extract proteins from olive bioresources because they are also valuable components [27].

### 3.6. Furfural and Hydroxymethylfurfural Amount in the EOP Extracts

The thermal degradation of carbohydrates in foods, like xylose and glucose, generates the undesirable compounds furfural and 5-hydroxymethylfurfural, respectively [28]. At high concentrations, these degradation compounds can reduce the food quality [29]. Considering that EOP contains 20.6% *w*/*w* polymeric carbohydrates and 1.8% free glucose [7], the concentrations of these sugar degradation products were determined in the extracts resulting from MAWE. Nevertheless, in the EOP aqueous extracts obtained in the BBD experiments, the concentrations of furfural and 5-hydroxymethylfurfural were lower than 0.012 g/L and 0.006 g/L, respectively. These values are very low with respect to another study on mandarin peels when subcritical water extraction was applied, which required more severe thermal conditions (130 °C, 20 min, 20% *w*/*v*) [28]. Therefore, these results highlight the fact that the thermal degradation of the EOP carbohydrates was negligible under the tested MAWE conditions.

### 3.7. Characterization of the EOP Solid after MAWE

#### 3.7.1. Chemical and Elemental Characterization

Aqueous extraction of EOP using microwave technology led to the elimination of a large part of the extractives [7]. As can be observed in Table 4, the raw EOP consisted of 41.8% extractives, whilst only 17.5% of the extractives was determined in the extracted EOP solid. MAWE solubilized about 75% aqueous extractives in raw EOP and consequently, the extracted EOP solid increased its ethanolic extractives content (from 3.8% up to 8.0%). This agrees with previous studies where an extraction step with water at 85 °C for 90 min [7] or 100 °C for 30 min [25] was applied to EOP. As a consequence of this solubilization of aqueous extractives, the percentage of hemicellulose and lignin in the extracted EOP solid increased by up to 13.5% and 31.5%, respectively, whilst the cellulose content barely changed (10.8%). This extracted EOP solid with 24.3% carbohydrates can be further exploited to recover free sugars from the polymeric fraction and added-value derivatives such as xylitol, bioethanol, lactic acid, etc., which would maximize the valorization of this bioresource. Moreover, the removal of extractives is also beneficial for the subsequent valorization process by promoting a better sugar recovery by enzymatic hydrolysis in cascading biorefinery approaches [25].

The elemental composition of the extracted EOP solid was also determined (Table 4). Compared to the raw EOP, a slight decrease in the nitrogen content was observed, i.e., from 1.3% to 1.1%. This fact indicates that a partial solubilization of protein occurred during the MAWE of biomass. This agrees with the presence of protein in the aqueous extract resulting from MAWE under optimized conditions, where 15.9 mg/g EOP was determined (Section 3.5).

#### 3.7.2. Scanning Electron Microscopy and Elemental Microanalysis

**Figure 3:**
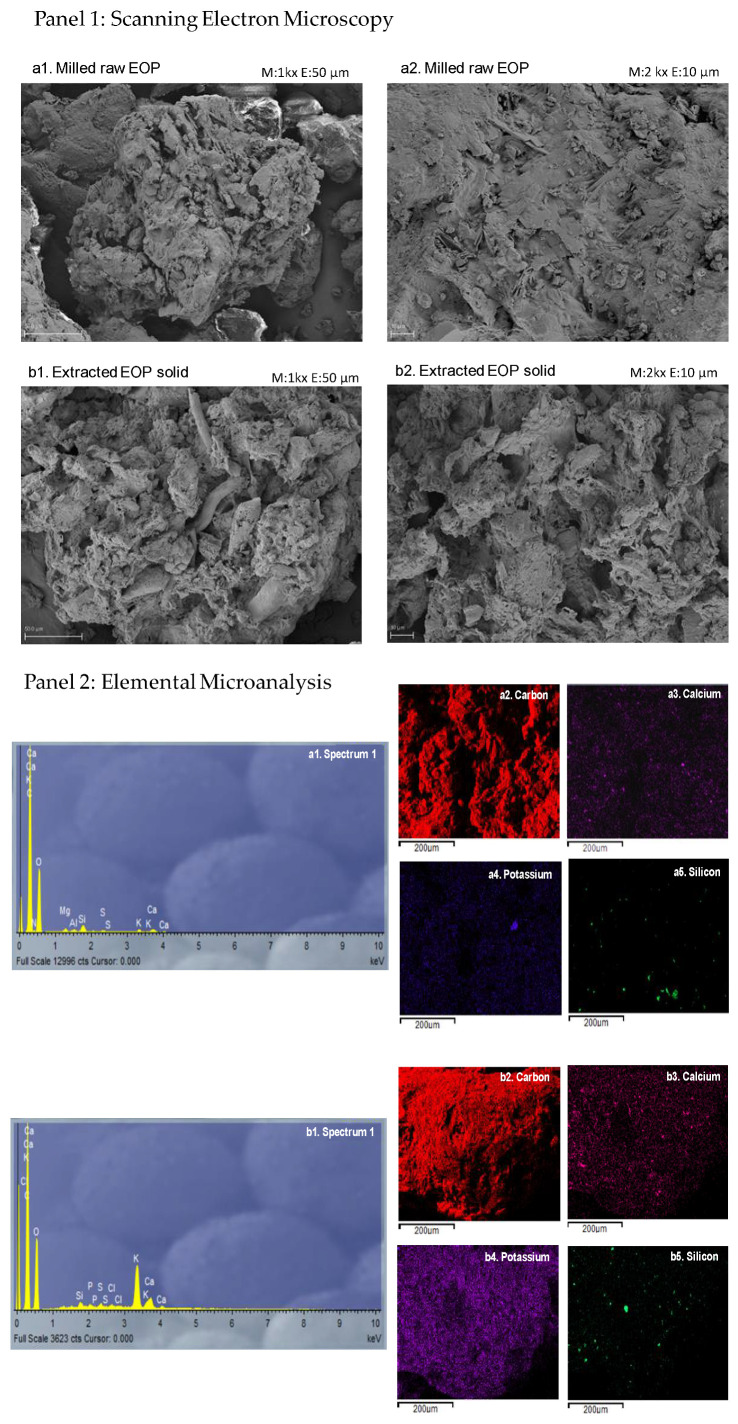
(**Panel 1**): Scanning electron microscopy images of the milled raw exhausted olive pomace (EOP) (**a1**,**a2**) and the extracted EOP solid obtained after microwave-assisted water extraction under optimized conditions (12% *w*/*v*, 100 °C, and 16 min) (**b1**,**b2**). (**Panel 2**): Spectrum and elemental maps of carbon, calcium, potassium, and silicon of the milled raw exhausted olive pomace (EOP) (**a1**–**a5**) and the extracted EOP solid (**b1**–**b5**) obtained by microwave-assisted water extraction at optimal conditions (12% *w*/*v*, 100 °C, and 16 min). The images were acquired by scanning electron microscopy–energy dispersive X-ray spectrometry.

The microscopic analysis of lignocellulosic biomasses such as EOP can provide an insight into the morphological alterations required for downstream biorefinery applications where the lignocellulosic structure should be broken. Therefore, to study the structural change that occurs due to the MAWE step, the extracted EOP solid was examined by SEM. Figure 3(a1,b1) (first panel) show panoramic images (50 µm scale) of the milled raw EOP and the extracted EOP solid, respectively. In raw EOP the olive fruit morphology was lost probably due to the overall processing suffering in the mill and the pomace olive oil extractor [30]. In both cases, plant material predominated, but in the extracted EOP solid, it was more fragmented and deteriorated. The MAWE effect was clearer when compared to Figure 3(a2,b2) (first panel) (at 10 µm scale). It can be appreciated that the extracted EOP solid presented more and larger holes. In this context, several authors observed that the use of MAE destroyed the sample surface. The sudden increase in temperature and internal pressure caused by the microwave irradiation affects the cell structure [31]. The structural changes provided by MAWE on EOP were slightly different from those found when ultrasound was applied, which promoted a frayed appearance to EOP [8]. The morphological change of the biomass is highly dependent on the technology and process conditions applied and more cracking lead to an increase in surface area and hydrolysis performance for downstream processing [32]. In general, it requires a cascading process to first obtain bioactive compounds in mild conditions as here to avoid their degradation and then more severe pretreatment conditions to favor the breakage of the lignocellulose structure to a higher extent to be valorized [18]. For example, previous studies on EOP have found that the hydrolysis performance of extracted EOP after ultrasound-assisted extraction for 12 min was lower than using a fractionation scheme based on an organosolv preatment (ethanol 50% *v*/*v* with sulfuric acid 1% *v*/*v*) at 130 °C for 60 min [9,18].

Furthermore, the SEM-EDX spectra showed that carbon, potassium, and calcium are major components (Figure 3(a1,b1), second panel), and these elements were distributed throughout the biomass before and after MAWE (Figure 3(a2–a4,b2–b4), second panel). Potassium is one of the major mineral components in the olive-derived biomasses [11]. Some studies suggest that the presence of potassium can limit the application of pomace as a biofuel for heating because it causes foiling in the boilers [33]. Alternatively, other studies suggest that the potassium-rich ash from biomasses such as olive pomace can be used as an alkaline activator in the production of sustainable geopolymers [34]. Therefore, besides being a source of sugars after pretreatment and hydrolysis [18], the latter application could be other ways to valorize EOP after the extraction of bioactive compounds by MAWE since potassium is still present.

### 3.8. Ethanolic Extraction of Triterpenic Acids

EOP also contains maslinic and oleanolic acids, whose extraction deserves attention due to their bioactive properties and potential uses in the pharmaceutical industry. As triterpenic acids were absent in the aqueous extracts, a second extraction was carried out to recover these compounds using ethanol due to the more apolar features of triterpenic acids compared with hydroxytyrosol and mannitol. This extraction step was carried out at room temperature as indicated in Section 2.6. Table 5 shows the results obtained after the ethanolic extraction of raw EOP and extracted EOP solid obtained by MAWE under optimized conditions. When the biomass had been previously extracted by MAWE, a higher maslinic acid concentration was determined, 0.53 g/L vs. 0.96 g/L. Likewise, when the ethanolic extraction was carried out after MAWE, the oleanolic acid concentration increased from 5.31 g/L up to 9.54 g/L. This fact can be related to the higher content of ethanolic extractives in the extracted EOP solid than in the raw EOP but also the effect of MAWE on the solid, which could enhance their extraction. In this sense, previous studies have shown that MAE applied to olive skin [20] and pomace [35] enhanced the extraction of triterpenic acids compared with Soxhlet extraction due to production of a higher tissue damage by the former.

Interestingly, the triterpenic acids concentrations determined in this work were higher than those obtained from olive pomace using MAE [36] and ultrasound-assisted extraction [37]. In this sense, olive leaves are considered a good source of this compound to produce high added-value products enriched in triterpenic acids [37,38]. The amount of maslinic acid extracted from EOP in this work is comparable to that reported by Martín-García et al. [38] from different varieties of olive leaves subjected to an ethanolic extraction (2.34–5.87 mg/g leaves). Therefore, EOP can also be further valorized to obtain these compounds.

In addition, the presence of residual hydroxytyrosol in the ethanolic extracts was evaluated and 0.17 mg HT/g EOP could still be recovered in the EOP solid extracted together with the triterpenic acids.

## 4. Conclusions

MAWE is an eco-friendly and efficient extraction for extracting polar bioactive compounds from EOP, such as hydroxytyrosol and mannitol. The extraction was influenced by the solid loading, extraction time, and temperature, and the results showed that intermediate values of the former (12% *w*/*v* and 16 min) together with a temperature of 100 °C maximized the overall responses. MAWE under optimized conditions resulted in an EOP antioxidant extract containing (per gram of EOP): 5.8 mg hydroxytyrosol, 46.7 mg mannitol, and 15.9 mg solubilized protein.

The MAWE step changed the EOP morphology and yielded solids enriched in ethanol extractives (8.1%), polymeric carbohydrates (24.3%), and lignin (31.5%). A second extraction step with ethanol allowed the extraction of triterpenic acids from the ethanolic extractives fraction, which was favored by the previous MAWE step: 9.54 and 3.60 mg/g extracted EOP solid, respectively. Therefore, it can be concluded that MAWE can be applied to efficiently recover bioactive compounds from EOP and this step can be complemented with other valorization options. In addition to obtaining olive pits and pomace-olive oil, the followed scheme applied to EOP could provide new incomes in the regional economy and a more sustainable scenario in the olive sector.

## Figures and Tables

**Table 1:** Experimental design showing the studied factors (temperature, time, and solid loading) and data obtained for the response variables.

Run	T	t	C	Yield	PC	TPC	HT	HTC	MAN	MANC	FRAP	ABTS
1	70	4	3	37.53	1.26	38.96	0.20	6.05	1.52	46.86	55.30	94.72
2	40	22	3	33.80	1.13	34.81	0.18	5.57	1.39	42.91	47.37	75.18
3	70	40	15	27.75	3.93	24.45	0.61	3.78	5.63	35.00	32.21	61.41
4	70	22	9	37.83	3.37	35.01	0.53	5.53	4.55	47.18	47.34	81.30
5	70	22	9	37.70	3.30	34.38	0.53	5.50	4.30	44.71	47.92	86.11
6	100	22	3	40.42	1.30	40.42	0.19	5.94	1.59	49.20	55.14	96.75
7	40	40	9	35.48	3.09	32.10	0.46	4.84	4.19	43.61	43.11	89.46
8	70	22	9	37.86	3.29	34.09	0.41	4.25	4.23	43.87	46.42	83.45
9	40	4	9	34.84	2.86	29.69	0.48	4.97	4.05	41.99	42.00	79.86
10	70	22	9	36.87	3.25	33.72	0.52	5.39	4.27	44.30	45.18	80.11
11	100	40	9	37.66	3.37	35.04	0.53	5.50	4.44	46.10	45.69	94.55
12	70	4	15	33.84	4.81	29.91	0.80	4.99	6.71	41.75	38.58	77.88
13	100	4	9	37.94	3.45	35.86	0.51	5.35	4.31	44.81	48.65	85.25
14	70	22	9	37.45	3.40	35.30	0.53	5.54	4.44	46.04	44.81	78.66
15	70	40	3	36.53	1.13	34.95	0.18	5.61	1.35	41.75	46.77	82.27
16	100	22	15	35.62	5.02	31.21	0.81	5.04	6.67	41.41	41.40	92.97
17	40	22	15	33.59	4.87	30.24	0.72	4.48	6.90	42.82	37.98	77.37

T, temperature (°C); t, extraction time (min); C, solid loading (%, *w*/*v*); yield (%); PC, phenolic concentration (g gallic acid equivalents/L), TPC, total phenolic content (mg gallic acid equivalents/g EOP), HT, hydroxytyrosol concentration (g/L), HTC, hydroxytytrosol content (mg/g EOP), MAN, mannitol concentration (g/L), MANC, mannitol content (mg/g EOP); antioxidant capacity (FRAP and ABTS) (mg Trolox equivalents/g EOP).

**Table 2:** Mathematical models (coded values) obtained in the Box-Behnken design for each response variable and statistical results.

Dependent Variable	Models	Eq.	CV (%)	R^2^	Adjusted R^2^	F-Value	Lack of Fit (*p*-Value)
Extraction yield (%)	37.54 + 1.74∙T + 0.093∙t − 1.25∙C − 0.23∙T∙t − 3.14∙t∙C + 1.37∙T^2^ − 2.43∙t^2^ − 3.06∙C^2^	(2)	1.64	0.9831	0.9577	38.77	0.1029
Phenolic concentration (g GAE/L)	3.32 + 0.15∙T + 0.034∙t + 1.87∙C − 0.075∙T∙t − 0.0057∙T∙C − 0.47∙t∙C + 0.23∙T^2^ − 0.36∙t^2^ − 0.47∙C^2^	(3)	2.93	0.9975	0.9939	270.38	0.0933
Total phenolic content (mg GAE/g EOP)	34.50 + 1.96∙T + 0.067∙t − 3.11∙C − 0.81∙T∙t − 1.16∙T∙C − 2.47∙t∙C + 1.44∙T^2^ − 2.77∙t^2^ − 1.77∙C^2^	(4)	2.53	0.9780	0.9449	29.57	0.1517
Hydroxytyrosol concentration (g/L)	0.53 + 0.025∙T − 0.00499∙t + 0.30∙C + 0.007083∙T∙t + 0.020∙T∙C − 0.087∙t∙C + 0.020∙T^2^ − 0.052∙t^2^ − 0.073∙C^2^	(5)	2.02	0.9991	0.9974	587.22	0.1473
Hydroxytyrosol content (mg/g EOP)	5.49 + 0.25∙T − 0.046∙t − 0.45∙C + 0.069∙T∙t + 0.048 T∙C − 0.51∙t∙C + 0.074∙T^2^ − 0.40∙t^2^ − 0.30∙C^2^	(6)	1.98	0.9871	0.9640	42.67	0.1484
Mannitol concentration (g/L)	4.35 + 0.058∙T + 0.042∙t + 2.67∙C − 0.004∙T∙t − 0.11∙T∙C − 0.56∙t∙C + 0.28∙T^2^ − 0.39∙t^2^ − 0.50∙C^2^	(7)	3.25	0.9974	0.9934	251.05	0.3651
Mannitol content (mg/g EOP)	44.73 + 1.27∙T + 0.73∙t − 1.97∙C − 0.081∙T∙t − 1.93∙T∙C − 4.10∙t∙C + 1.57∙T^2^ − 2.17∙t^2^ − 2.21∙C^2^	(8)	1.88	0.9799	0.9346	21.65	0.8828
FRAP (mg TE/g EOP)	46.33 + 2.55∙T − 0.77∙t − 5.48∙C − 1.02∙T∙t − 1.08∙T∙C − 2.11∙t∙C + 1.72∙T^2^ − 3.19∙t^2^ − 2.58∙C^2^	(9)	2.78	0.9779	0.9448	29.51	0.6168
ABTS (mg TE/g EOP)	81.92+ 3.24∙T + 5.35∙t + 4.40∙C − 0.076∙T∙t − 6.92∙T∙C − 14.21∙t∙C + 15.24∙T^2^ − 9.88∙t^2^ − 6.17∙C^2^	(10)	3.55	0.9595	0.8865	13.15	0.3543

T, temperature (°C); t, extraction time (min); C, solid loading (%, *w*/*v*).

**Table 3:** Predicted results obtained by the model at optimal conditions (12% *w*/*v*, 100 °C, and 16 min) and experimental results measured after the application of these conditions. Data represent the mean value and standard deviation (n = 5).

Response Variable	Predicted Values	Experimental Values	Error (%)
Extraction Yield (%)	39.22	39.15 ± 2.40	0.18
Phenolic concentration (g GAE/L)	4.48	4.30 ± 0.17	4.20
TPC (mg GAE/g EOP)	35.95	34.49 ± 1.38	4.20
Hydroxytyrosol concentration (g/L)	0.71	0.73 ± 0.03	2.74
Hydroxytyrosol content (mg/g EOP)	5.59	5.87 ± 0.28	4.77
Mannitol concentration (g/L)	5.76	5.83 ± 0.13	1.20
Mannitol content (mg/g EOP)	45.5	46.70 ± 1.09	2.51
FRAP (mg TE/g EOP)	47.17	45.35 ± 2.09	4.01
ABTS (mg TE/g EOP)	97.36	98.78 ± 1.54	1.43

GAE, gallic acid equivalents; TE, Trolox equivalents.

**Table 4:** Chemical composition of the raw exhausted olive pomace (EOP) and extracted EOP solids obtained after microwave-assisted water extraction under optimized conditions (12% solids, 100 °C, and 16 min). Data are expressed as mean value and standard deviation (n = 3 for EOP and n = 5 extracted for EOP solids).

Component	Raw EOP ^1^	Extracted EOP Solid
Chemical characterization	%, dry weight basis	%, dry weight basis
Extractives	41.8 ± 1.9 ^a^	17.5 ± 0.9 ^b^
Aqueous extractives	37.9 ± 1.9 ^a^	9.5 ± 1.0 ^b^
Ethanolic extractives	3.8 ± 0.2 ^b^	8.1 ± 0.1 ^a^
Cellulose	9.7 ± 0.8 ^a^	10.8 ± 1.3 ^a^
Hemicellulose	10.9 ± 0.5 ^b^	13.5 ± 1.0 ^a^
Xylose	9.8 ± 0.5 ^b^	13.1 ± 1.4 ^a^
Galactose	0.3 ± 0.0 ^b^	1.1 ± 0.3 ^a^
Arabinose	1.8 ± 0.0 ^a^	1.0 ± 0.1 ^b^
Mannose	0.4 ± 0.0	-
Acetyl groups	1.5 ± 0.2 ^a^	1.3 ± 0.3 ^a^
Lignin	21.8 ± 0.9 ^b^	31.5 ± 0.5 ^a^
Ash	6.4 ± 0.2 ^a^	1.4 ± 0.0 ^b^
Elemental analysis	%, dry weight basis	%, dry weight basis
Nitrogen	1.3 ± 0.1 ^a^	1.1 ± 0.2 ^a^
Carbon	42.4 ± 0.2 ^b^	49.7 ± 0.2 ^a^
Hydrogen	5.6 ± 0.1 ^b^	6.1 ± 0.0 ^a^
Sulfur	ND	1.7 ± 0.0

ND: not detected. ^1^ Gómez-Cruz et al. [18]. In each row, different superscript letters denote significant differences between the means (*t*-test, *p* < 0.05).

**Table 5:** Triterpenic acids extracted from the milled raw exhausted olive pomace (EOP) and extracted EOP solid obtained by microwave-assisted water extraction at optimal conditions (12% *w*/*v*, 100 °C, and 16 min).

Parameter	Raw EOP	Extracted EOP Solid
Extraction yield (%, g/100 g solid)	6.40 ± 0.19 ^b^	6.78 ± 0.14 ^a^
Maslinic acid concentration (g/L)	0.53 ± 0.01 ^b^	0.96 ± 0.01 ^a^
Maslinic acid content (mg/g solid)	5.31 ± 0.12 ^b^	9.54 ± 0.03 ^a^
Oleanolic acid concentration (g/L)	0.20 ± 0.01 ^b^	0.36 ± 0.00 ^a^
Oleanolic acid content (mg/g solid)	1.96 ± 0.05 ^b^	3.60 ± 0.02 ^a^

In each row, different superscript letters denote significant differences between the means (*t*-test, *p* < 0.05).

## Data Availability

Data is contained within the article.

## Supplementary Materials

The following supporting information can be downloaded at: https://www.mdpi.com/article/10.3390/foods11142002/s1, Table S1: Retention time, *m*/*z* of the molecular ion and fragmentation pattern of the compounds characterized in the aqueous extract from exhausted olive pomace obtained by microwave-assisted water extraction at optimal conditions; Table S2: Experimental values for the protein solubilized in the aqueous extracts obtained in the Box-Behnken design experiments; Table S3: Statistical parameter values for the factors of the Box-Behnken design applied to the exhausted olive pomace (EOP) and in the response of the protein solubilized in the aqueous extracts obtained by microwave-assisted water extraction; Figure S1: HPLC chromatogram at 280 nm of the aqueous extract of extracted olive pomace obtained by microwave-assisted water extraction at optimal conditions (12%, *w*/*v*, 100 °C, and 16 min)**.**
