# Supplementary material for: Optimization of Microwave-Assisted Water Extraction to Obtain High Value-Added Compounds from Exhausted Olive Pomace in a Biorefinery Context
Source: Foods. 2022 Jul 6;11(14):2002. doi: 10.3390/foods11142002 (PMC9320046; doi:10.3390/foods11142002)
Supplement: Supplementary file 1 [file foods-11-02002-s001.zip › foods-1763391-supplementary.pdf]

foods

Article

Optimization of Microwave-Assisted Water Extraction to Obtain High Value-Added Compounds from Exhausted Olive Pomace in a Biorefinery Context

Irene Gómez-Cruz [1,2], María del Mar Contreras [1,2,*], Inmaculada Romero [1,2] and Eulogio Castro [1,2]

[1] Department of Chemical, Environmental and Materials Engineering, Universidad de Jaén, Campus Las Lagunillas, S/N, 23071 Jaén, Spain; igcruz@ujaen.es (I.G.-C.); iromero@ujaen.es (I.R.); ecastro@ujaen.es (E.C.)
[2] Centre for Advanced Studies in Earth Sciences, Energy and Environment (CEACTEMA), Universidad de Jaén, Campus Las Lagunillas, S/N, 23071 Jaén, Spain
* Correspondence: mcgamez@ujaen.es

Supplementary materials: 1 Figure and 3 Tables

Citation: Gómez-Cruz, I.; Contreras, M.d.M.; Romero, I.; Castro, E. Optimization of Microwave-Assisted Water Extraction to Obtain High Value-Added Compounds from Exhausted Olive Pomace in A Biorefinery Context.. *Foods* **2022**, *11*, 2002. https://doi.org/10.3390/foods11142002

Academic Editor: Antonello Santini

Received: 24 May 2022
Accepted: 4 July 2022
Published: 6 July 2022

Publisher's Note: MDPI stays neutral with regard to jurisdictional claims in published maps and institutional affiliations.

Copyright: © 2022 by the authors. Licensee MDPI, Basel, Switzerland. This article is an open access article distributed under the terms and conditions of the Creative Commons Attribution (CC BY) license (https://creativecommons.org/licenses/by/4.0/).

Figures

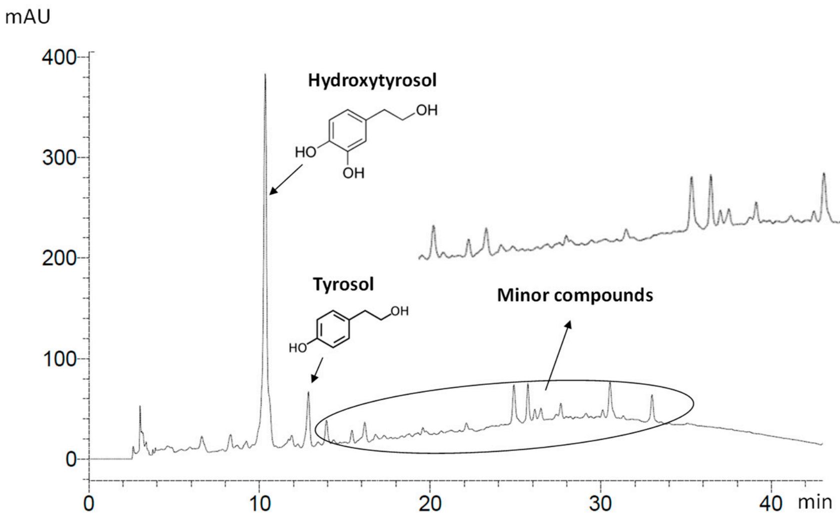

Figure S1. HPLC chromatogram at 280 nm of the aqueous extract of extracted olive pomace obtained by microwave-assisted water extraction at optimal conditions (12%, w/v, 100 ºC, and 16 min).

Tables

Table S1. Retention time, *m*/*z* of the molecular ion and fragmentation pattern of the compounds characterized in the aqueous extract from exhausted olive pomace obtained by microwave-assisted water extraction at optimal conditions.

Nº	RT (min)	$[M-H]^-$ (*m*/*z*)	Fragments (*m*/*z*)	Compound
	Hydroxytyrosol derivatives			
1	1.1	153	123	Hydroxytyrosol[1]
2	1.2	315	153, 135, 123	Hydroxytyrosol glucoside
3	1.7	461	315, 161, 135	Verbasoside
4	1.9	299	179, 161, 119, 101	Tyrosol glucoside
5	3.0	461	299, 179, 161	Verbasoside isomer
7	6.5	195	153, 151, 59	Hydroxytyrosol acetate
8	9.3	483	347, 123	Oleacein derivative (+ hexose + H_2)
10	9.9	543	513, 377, 357, 313	Dihydro oleuropein
11	10.1	623	461, 315	Verbascoside
13	10.3	701	539, 437, 377, 307, 275	Oleuropein hexoside isomer 1
16	10.7	335	317, 199, 153	Hydroxy oleacein isomer 1
17	10.8	701	539, 377, 307, 275	Oleuropein hexoside isomer 2
18	10.9	623	461	Isoverbascoside
19	11.0	335	317, 199, 153, 111	Hydroxy oleacein isomer 2
20	11.3	541	361, 225, 181	Oleuropein derivative (+H_2)
21	11.5	539	403, 223	Oleouropein isomer 1
23	11.9	539	403, 377, 307, 275, 223	Oleuropein[1]
24	11.9	701	377, 307, 275	Oleuropein hexoside isomer 3
25	12.5	539	377, 307, 275, 223	Oleouropein isomer 2
26	12.9	539	403, 377, 307, 275, 223	Oleouropein isomer 3
27	13.0	319	183, 181, 153, 111	3,4-DHPEA-EDA[2] or oleacein
29	13.6	523	361, 291, 259, 223	Ligustroside
	Other phenolic compounds			
6	6.2	137	Not fragmented	3-Hydroxybenzoic acid
9	9.7	463	347, 301	Quercetin glucoside
12	10.3	447	285	Luteolin 7-*O*-glucoside
14	10.3	593	285	Luteolin *O*-deoxyhexosyl-hexoside
15	10.7	593	447, 285	Luteolin *O*-deoxyhexoside *O*-hexoside
22	11.7	551	507, 389, 341, 281, 251, 179, 161	Caffeoyl-6'-secologanoside
28	13.0	535	491, 389, 345, 265, 163	*p*-Coumaroyl-6'-secologanoside
	Other compounds			
a	0.4	191	111	Citric acid

b	0.5	191		Quinic acid
c	0.5	181	163, 143, 131, 119, 113	Mannitol
d	0.8	393	375, 213, 151	Unknown
e	1.5	407	389, 375, 357, 313	Acyclodihydroelenolic acid hexoside
f	2.2	553	491, 371, 181	Unknown
g	3.3	183	139	Decarboxymethylelenolic acid
h	4.0	389	345, 209, 165, 121	Oleoside/Secologanoside
i	4.4	435	389, 313, 357, 161	Acyclodihydroelenolic acid hexoside derivative (+CO)
j	5.4	377	197, 153	Elenolic acid derivative
k	5.8	389	357, 313	Acyclodihydroelenolic acid hexoside derivative (-H_2, -O)
l	14.4	836	790.0	Unknown
m	15.5	557	513, 345, 209	6'-*O*-[(2E)-2,6-Dimethyl-8-hydroxy-2-octenoyloxy]-secologanoside
n	16.7	329	311, 275, 201, 171	Trihydroxyoctadecenoic acid
ñ	18.1	331	313, 295, 171	Trihydroxyoctadecanoic acid
o	19.0	287	269	Dihydroxyhexadecanoic acid

[1]Compared with standards. [2]Oleacein or 3,4-DHPEA-EDA or decarboxymethyl oleuropein aglycone.

Table S2. Experimental values for the protein solubilized in the aqueous extracts obtained in the Box-Behnken design experiments.

RUN	T (ºC)	t (min)	C (%, w/v)	g BSA/L	mg BSA/g EOP
1	70	4	3	0.40	12.37
2	40	22	3	0.19	5.76
3	70	40	15	1.52	9.43
4	70	22	9	1.68	17.43
5	70	22	9	1.68	17.50
6	100	22	3	0.51	15.76
7	40	40	9	1.31	13.57
8	70	22	9	1.16	12.00
9	40	4	9	1.19	12.38
10	70	22	9	1.30	13.53
11	100	40	9	1.29	13.39
12	70	4	15	2.03	12.64
13	100	4	9	1.58	16.47
14	70	22	9	1.69	17.59
15	70	40	3	0.35	10.72
16	100	22	15	2.56	15.89
17	40	22	15	2.02	12.52

Table S3. Statistical parameter values for the factors of the Box-Behnken design applied to the exhausted olive pomace (EOP) and in the response of the protein solubilized in the aqueous extracts obtained by microwave-assisted water extraction.

	Protein (g/L)		Protein (mg/g EOP)	
Source	F-Ratio	P-Value	F-Ratio	P-Value
T: Temperature	2.940	0.130	5.410	0.053
t: Time	1.100	0.330	0.830	0.394
C: Solids	86.020	<0.0001	0.620	0.456
TT	0.110	0.746	0.033	0.862
Tt	0.640	0.451	0.660	0.442
TC	0.190	0.680	1.600	0.247
tt	2.660	0.147	1.240	0.302
tC	0.820	0.395	0.088	0.775
CC	3.370	0.109	5.130	0.058
